# Editorial: *C. elegans* as an emerging model of pharmacological innovation

**DOI:** 10.3389/fphar.2022.1029752

**Published:** 2022-09-27

**Authors:** Zebo Huang, Long Ma, Ajay Mishra, Jeremy E. Turnbull, Haijun Tu

**Affiliations:** ^1^ Guangdong Province Key Laboratory for Biocosmetics, School of Food Science and Engineering, South China University of Technology, Guangzhou, China; ^2^ School of Life Sciences, Central South University, Changsha, China; ^3^ European Bioinformatics Institute (EMBL-EBI), Cambridge, United Kingdom; ^4^ Centre for Glycoscience, School of Life Sciences, Keele University, Liverpool, United Kingdom; ^5^ College of Biology, Hunan University, Changsha, China

**Keywords:** disease modeling, drug discovery, aging, lifespan, healthspan, neurodegeneration, proteotoxicity, oxidative stress

As a model organism, the genetically tractable nematode *Caenorhabditis elegans* has helped revolutionize modern life sciences, leading to the pioneering work of programmed cell death, RNA interference (RNAi) and green fluorescent protein (GFP) and the wide-range applications of such technologies in biomedical areas. In the past decade, *C. elegans* has been increasingly used as a favorable and affordable *in vivo* animal model in pharmacological research and drug discovery, including target as well as lead identification. Indeed, *C. elegans* has already shown great power in the evaluation of potential therapeutics at both molecular and organismal levels owing to its short lifespan, relative simplicity and high degree of experimental tractability, which is further strengthened by significant conservation of disease genes and signaling pathways with humans. For example, transgenic *C. elegans* strains expressing human disease proteins have been generated to model neurodegenerative disorders such as Alzheimer’s, Parkinson’s and Huntington’s diseases, and have been used to screen for pharmacologically active compounds and to study their mechanisms of action (Wang et al., 2021; Egan et al.; Wang and Zheng). These studies not only exemplify the advantage of *C. elegans* in animal-based high-throughput screening but also provide an insight into the mechanisms of candidate compounds, demonstrating its transformative power in translational research for drug discovery and development. Due to its rapid life cycle and short lifespan, *C. elegans* can also be used to monitor long-term and even cross-generational outcomes of pharmacological interventions. Furthermore, *C. elegans* can also be conveniently used to study the overall efficacy, multitargeting capacity and potential interactions of component herbs of traditional complex formulae as recently demonstrated by formula dissection approaches (Xiao et al., 2020). However, to fully unleash the potential of *C. elegans* in pharmacology, methodological, strategic and application innovations are further needed.

Aging is a major risk factor for many age-related diseases in humans, including late-onset neurodegenerative diseases. Thus, anti-aging interventions and lifespan-control mechanisms are likely to play a role in preventing age-related pathogenesis (Egan et al.; Xiao et al., 2022). The renin-angiotensin system (RAS) is an endocrine system, which is often a target for reducing blood pressure by drugs such as angiotensin-converting enzyme (ACE) inhibitors. Interestingly, polymorphisms in the ACE gene are associated with longevity in humans, and decrease of ACN-1 (the ACE homolog) activity is shown to extend lifespan of *C. elegans*, suggesting a conserved role of RAS in longevity regulation. Egan et al. made a timely review on studies of RAS and aging with respect to the potential of *C. elegans* as a model to explore lifespan control mechanisms. Tan et al. reported that complanatoside A, a flavonol glycoside isolated from the seed of the herbal plant *Astragalus complanatus*, was able to increase stress resistance, extend lifespan, enhance locomotor capacity and reduce accumulation of aging pigments of *C. elegans*, demonstrating its stress and healthspan regulating effects. Further investigations revealed that complanatoside A was also able to reduce the accumulation of amyloid β-peptide (Aβ) and α-synuclein and delay the onset of associated neurodegeneration in *C. elegans* models (Tan et al.). The authors then showed that the lifespan-extending effect of complanatoside A was correlated with DAF-16/FOXO, SKN-1/Nrf2 and HSF-1. Interestingly, earlier studies have reported that astragalan, a polysaccharide isolated from the roots of *Astragalus membranaceus*, is able to alleviate neurotoxicity mediated by polyglutamine (polyQ) and extend lifespan of *C. elegans* in a DAF-16/FOXO-dependent fashion (Zhang et al., 2012). Astragalan has also been shown to suppress 6-hydroxydopamine-induced neurotoxicity in *C. elegans* involving alleviation of oxidative stress, regulation of the apoptosis pathway and restoration of cholinergic system function (Li et al., 2016). Together, these findings suggest that stress and lifespan regulators have a great potential in the prevention of proteotoxic diseases.

Andersen et al. explored novel functions of imidazolium salts by using imidazole rings as scaffolds and using *C. elegans* as *in vivo* models with the aim of developing proteotoxic and neurodegenerative therapeutics. Among various imidazolium salts, 1-mesityl-3-(3-sulfonatopropyl) imidazolium was identified to be effective against iron-induced oxidative stress in *C. elegans*, and the protective effect was shown to be associated with the heat shock transcription factor HSF-1 (Andersen et al.). The compound was further found to reduce the proteotoxicity in *C. elegans* models expressing the pathogenic proteins Aβ, α-synuclein or polyQ, including amelioration of Alzheimer’s disease-like paralysis and delay of age-related locomotion decline (Andersen et al.). These broad neuroprotective effects provide an important insight into the anti-neurodegenerative potential of the compound *per se* and a proof of concept for promising development of imidazole derivatives as novel therapies of age-related proteotoxic diseases. On the other hand, Mohankumar et al. found that α- and β-santalol, the natural sesquiterpenes from sandalwood, were able to stabilize the native state and potently inhibit the aggregation of transthyretin (TTR), another type of pathological proteins implicated in inherited amyloidosis. Specifically, the santalol isomers were shown to reduce wild-type and mutant TTR aggregates in *C. elegans* models expressing TTR fragments in body wall muscle cells and also extend both lifespan and healthspan of the nematodes by activating SKN-1/Nrf2, autophagy and proteasome pathways. Collectively these findings demonstrates their potential as therapeutic intervention against TTR amyloidogenesis and associated diseases (Mohankumar et al.).

Over recent years, it has emerged that the interactions between gut microbiome and the brain play an important role in neurodegeneration but when and how it happens remain largely unknown. Wang and Zheng highlighted the advantages of *C. elegans* models in unravelling the microbe-host interactions that may regulate neurodegeneration, and identified pro- as well as anti-neurodegenerative microbial factors, including bacterial amyloid proteins. Therefore, targeting microbe-host interactions, e.g. targeting production of amyloid-like proteins in gut bacteria, is a potentially novel strategy in the discovery of neurodegenerative interventions as demonstrated in their screening work. In this regard, *C. elegans* can be conveniently used as animal models in high-throughput screening platforms for potential neurodegenerative therapeutics that target the microbe-host interactions (Wang and Zheng).

In summary, the article collection in the research topic “*C. elegans* as an Emerging Model of Pharmacological Innovation” provided timely updates and state-of-the-art strategies through the expertise of contributing authors to explore the advantages of *C. elegans* as an alternative animal model in pharmacology. It is also our hope that this dedicated topic provides useful information and demonstrates important aspects justifying a broader use of this model animal in pharmacological research, including aging pharmacology, neuropharmacology, behavioral pharmacology, metabolic pharmacology, gut-brain interactions, pharmacogenomics, epigenetics and epigenome, target discovery, drug discovery, and ethnopharmacology.
